# Sclerosing Xanthofibroma of the Rib That Mimics a Very Aggressive Malignant Tumor of the Thoracic Wall

**DOI:** 10.1155/2014/894167

**Published:** 2014-09-23

**Authors:** F. Caushi, D. Xhemalaj, I. Bani, I. Skenduli, B. Gega, H. Hafizi, A. Mezini

**Affiliations:** ^1^Department of Thoracic Surgery, University Hospital “Shefqet Ndroqi”, Street “Shefqet Ndroqi”, Tirana, Albania; ^2^Department of Pathologic Anatomy, University Hospital “Shefqet Ndroqi”, Tirana, Albania; ^3^Department of Pathology, University Hospital “Shefqet Ndroqi”, Tirana, Albania; ^4^Department of Radiology, University Hospital “Shefqet Ndroqi”, Tirana, Albania

## Abstract

Sclerosing xanthofibroma is a benign lesion generally of flat bones that is thought to be caused by a reactive response to intramedullary hemorrhage following chest wall trauma. We are reporting a case of a 56-year-old man that was complaining of a dump pain on the right back and a swelling right in this place for several weeks. The radiology was suggesting an aggressive malignant tumor of the chest wall and probably metastasis in both lungs meanwhile the patient was in good state and very active. The surgery was decisive for the diagnosis that, to the fortune of the patient, it was sclerosing xanthofibroma.

## 1. Introduction

The tumors of thoracic wall are numerous and vary from benign lesions to very aggressive malignant ones with a poor prognosis. In most of them the radiology is very helpful for the diagnosis, but the biopsy is the most decisive diagnostic procedure. One of the benign lesions of the thoracic wall that is rarely found is sclerosing xanthofibroma of the rib [1–7].

## 2. Case Report

This is the case of a 56-year-old man that was complaining of a dump pain on the right back and a swelling right in this place for several weeks. The patient was in good state and very active. There was not any health problem in the past except a thoracic trauma at work one year ago. In that time the patient was diagnosed with a simple fracture of the 9th right rib without any other consequences.

**Figure 1:**
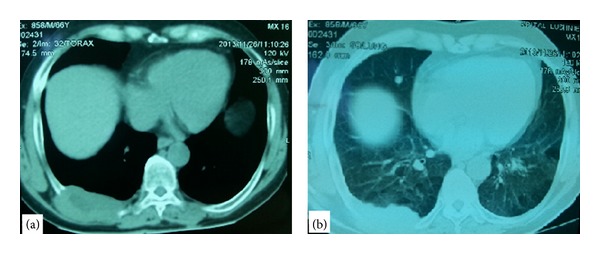
CT-scan. (a) Chest window that shows the tumor of thoracic wall. (b) Lung window that shows a node in the lower lobe of the right lung besides the tumor of thoracic wall.

On the X-ray was seen a shadow in the lower part of the right hemithorax. After that, it was decided to perform a CT-scan of the thorax that revealed a tumor of the thoracic wall in the right hemithorax that measured 8 × 4 cm and had a heterogeneous density inside of it. The tumor had involved and destructed the 9th rib and was lying even in two adjacent intercostal spaces, but without involving the lung and muscular layers. On lung window of the CT-scan were seen micronodular infiltrations of both lungs with diameters up to 5 mm and only one nodule in lower lobe of the right lung with diameter almost 1 cm (Figure 1). The mediastinum was without enlarged lymph nodes and no other lesion was seen in adjacent organs. The conclusion of the radiologist was that probably this was the case of a malignant tumor of the thoracic wall with secondary lesions in both lungs and a biopsy of the tumor was recommended.

**Figure 2:**
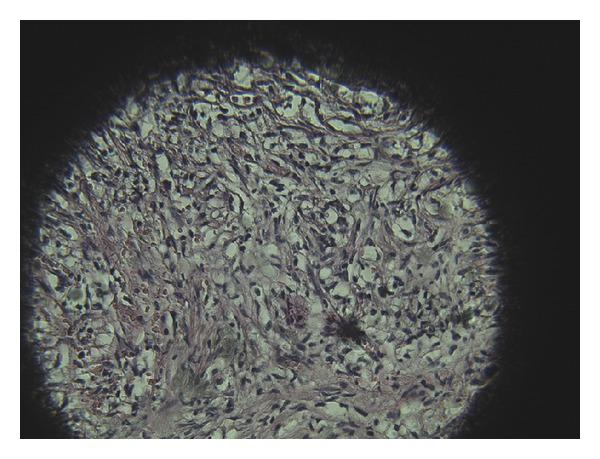
There are xanthomatous cells separated from fibrotic septae in a fibrotic stroma, without atypia and no cellular pleomorphism.

Because there was not a correlation between the clinical picture of the patient and the conclusion of the radiology it was decided to perform frozen biopsy of the lesions of the right lung and an excision biopsy of the tumor of the thoracic wall. So, through a small posterior thoracotomy at 7th intercostal space were sampled five nodular lesions from the right lung and a sample from the tumor. None of these samples resulted positive (no malignant cells found) after frozen biopsy. In such conditions was considered as more realistic the option of performing an oncologic resection of the tumor of the thoracic wall. (We resected three ribs where the 8th and 10th ribs were macroscopically free of the tumor. The resection was extended 10 cm anteriorly from the tumor and posteriorly it was accompanied by disarticulation of the ribs and partial resection of transversal processes of 8th, 9th, and 10th vertebras.) After that, we have performed a plastic procedure with polypropylene mesh in double layers to correct the defect. The clinical course of the patient was very good and five days later he was discharged from the hospital in very good condition. The conclusion of biopsy for both the tumor of thoracic wall and the lung nodules was sclerosing xanthofibroma which is considered by most people to be a tumor with different clinical and radiological features. Lesions were characterized by a network of anastomosing bone trabeculae without osteoblast lining within a fibrous stroma (Figure 2).

Referring to the follow-up, the patient was in a great state of health and from the first month after the surgery he turned back at his work place. Two years after the surgery the thoracic CT-scan showed neither recurrences of the lesions of the thoracic wall nor new developments on the lungs.

## 3. Discussion

The exact nature of sclerosing xanthofibroma remains controversial. Most of authors have considered being developmental lesions rather than true neoplasms. For some authors it is considered as a benign variant of malignant fibrous histiocytoma. The lesion is believed to be a reactive response to intramedullary hemorrhage following chest wall trauma. Almost all the reported cases of sclerosing xanthofibroma have occurred in patients older than 20 years of age and most of them were accompanied by pain. The lesions in sclerosing xanthofibroma are described as lytic, most with sharply defined margins and a sclerotic rim. The lesions are small and most commonly involve the flat bones, especially the pelvis or ribs. In long bones, the metaphysis, epiphysis, or diaphysis may be involved. In 70 cases of sclerosing xanthofibroma known and reported in a study of Blanco et al. only six of them belong to the rib [1]. Cortical expansion and soft tissue invasion as in our case have been reported only rarely. Sclerosing xanthofibroma is behaving in an aggressive manner with a potential for local spread and distant dissemination and has a tendency to recur after curettage [1–7].

The differential diagnosis of sclerosing xanthofibroma has to be done with nonossifying fibroma and fibrous cortical defect that are not true neoplasms, only developmental defects. The differential diagnosis has to be done even with giant cell tumor, giant cell reparative granuloma, eosinophilic granuloma, osteosarcoma, and so forth [1–7].

## 4. Conclusion

Sclerosing xanthofibroma of the rib is a rare tumor of the chest wall that radiologically may resemble very much a malign tumor but does not behave as it.

The clinical status of the patient that does not show such a great sufferance having a suspected malign tumor and the history of a trauma of the thoracic wall must not be ignored. However the clue of the diagnosis for sclerosing xanthofibroma is the biopsy.

Although according to the literature the recurrences do not happen after a partial resection, we believe that when the diagnosis is not sure and rare as in our case, at least the resection of the interested rib is necessary for a clear diagnosis and for the releasing of the pain.
